# Older Users of a Befriending Service in Ireland and the Maintenance of Personal Autonomy during the COVID-19 Pandemic

**DOI:** 10.3390/ijerph18062788

**Published:** 2021-03-10

**Authors:** Eimile Holton, Rachel Fitzpatrick, Rebecca Maguire, Seán Commins, Thomas Scharf, Brian Lawlor, Natalie Johnson, Caoimhe Hannigan, Joanna McHugh Power

**Affiliations:** 1 School of Medicine, Trinity College Dublin, Dublin 2 Dublin, Ireland; eholton@tcd.ie (E.H.); blawlor@stjames.ie (B.L.); Caoimhe.Hannigan@ncirl.ie (C.H.); 2 Department of Psychology, Maynooth University, Maynooth, Co. Kildare, Ireland; Rachel.Fitzpatrick.2019@mumail.ie (R.F.); Rebecca.Maguire@mu.ie (R.M.); Sean.Commins@mu.ie (S.C.); 3 Health Sciences Institute, Newcastle University, Newcastle NE1 7RU, UK; Thomas.Scharf@newcastle.ac.uk; 4 ALONE, Dublin 8 Dublin, Ireland; Natalie.Johnson@alone.ie; 5 National College, Ireland School of Business, Dublin 1 Dublin, Ireland

**Keywords:** befriending, COVID-19, public health, grounded theory, older adults

## Abstract

In the Republic of Ireland (RoI), COVID-19 public health guidelines have been most restrictive for people aged 70 and over. Such individuals are most likely to avail of befriending services offered by a network of Irish organisations. The aim of this study was to explore the impact of COVID-19 guidelines on befriending service users, and to develop recommended adaptations to befriending services compatible with such guidelines. A qualitative constructivist grounded theory approach was taken to the study design and analysis, using semi-structured interviews to collect data from 11 participants by telephone between May 2020 and January 2021. Results show a grounded theory describing how older users of a befriending service maintained their personal autonomy in the face of strict government guidelines. Participants described living life as usual, often contravening guidelines, and how they chose to adapt to the situation, yielding both positive and negative outcomes. Some potential adaptations were discussed to the befriending service (including a preserved focus on the social and emotional functions of the befriending relationship, and the accommodation of collaborative decision making about communicative alternatives), but ultimately it was made clear that participants would tailor the services to their own preferences. Results have implications for befriending service design and delivery, and for public health officials who wish to support the health of older adults during the COVID-19 pandemic.

## 1. Introduction

“Lockdowns” and stay-at-home orders have characterised the international response to the COVID-19 pandemic of 2020, particularly for people aged 70 and over, and for the medically vulnerable. The Republic of Ireland (RoI) saw its first confirmed case of COVID-19 in February 2020, and within one month had accrued 2615 cases. Alongside other public health measures, the Irish government advised, from 27 March 2020, that citizens aged 70 and over remain at home (“cocooning”), avoiding unnecessary interaction with non-household members. Similar measures were taken in other countries, including the UK (BBC, 2020). In RoI, cocooning measures remained in place until 18 May 2020, and were re-issued in June 2020, October 2020, and again in December 2020. 

Such measures were introduced primarily to protect the physical health of people aged 70 and over. However, a significant proportion of this age group experience other critical issues. Notably, 32% of people aged 75 and over are moderately isolated, with 8% highly isolated, based on nationally representative data [1]. While aimed at protecting physical health, cocooning is likely to exacerbate such isolation in this age group, particularly among people living alone [2]. Social isolation and relatedly loneliness are both undesirable in their own right, but also because they constitute risk for other health issues, such as cognitive and cardiovascular decline and early mortality [3,4,5,6]. In a recent survey of 150 Irish adults aged 70 and over, 40% reported worse or much worse mental health as a result of cocooning, with 1 in 8 reporting that they felt lonely very often [7]. Among people aged 60 and over, scores of loneliness more than doubled between 2019 and 2020 [8]. 

It is worth noting that, while the increased social isolation brought on by COVID-19 was expected to exacerbate mental health issues among the general older population [9], this has not reliably transpired. While concern was initially raised about the mental health impact of the pandemic, for all age groups, the sharpest rises in depressive and anxiety symptoms occurred in the immediate aftermath of its onset, and decreased steadily thereafter [10]. Additionally, while loneliness has increased in the older population since the beginning of the pandemic [8,11] the increase was greater among younger adults [12]. Similarly, a comparison of risk factors for loneliness showed that only individuals aged 18–30 had increased risk of loneliness during, relative to before, the pandemic [13], indicating that age moderated the impact of the pandemic on loneliness. This unexpected finding may be related to superior resilience in later life [12], or it may be a function of the “loneliness threshold” which frames loneliness as a comparative exercise [14]. It is possible that older adults had a lower threshold of social interaction prior to the pandemic, meaning that the restrictions brought about less disruption in this group than in the younger generation, whose social interactions were more severely curtailed. Similar patterns of preserved wellbeing for older people were found for the outcomes of psychological distress, negative affect, depression, and anxiety, in German and U.S. populations [15,16]. 

That said, concern remains about people who typically use services to reduce social isolation and loneliness, since such services have been disrupted by cocooning measures [2]. One such service is befriending—formal visiting arrangements typically mediated by a third party organization [17]. Across RoI, more than 60 befriending services are connected by the Befriending Network Ireland, run by the charity organization ALONE (www.alone.ie/befriending-networks-ireland, accessed on 7 March 2021). The stated aim of these services is to reduce the impact of loneliness and social isolation on the health of older adults [18]. ALONE befriending services pair voluntary befrienders with service users in a structured manner, typically involving weekly face-to-face visits, or sometimes telephone calls. While befriending in its face-to-face format is precluded by cocooning guidelines, it has been recommended specifically as a potential loneliness intervention during the pandemic [19].

In response to the cocooning guidelines issued in March 2020, ALONE requested that all face-to-face visits in their befriending services cease, and advised instead that telephone or videoconferencing methods be used. Similarly, Befriending Networks UK advised their members to avoid face-to-face contact and use other remote methods of connecting (http://www.befriending.co.uk/news/coronavirus-09032020, accessed on 7 March 2021). While there are concerns about the accessibility of such remote options for older adults, they do appear to be acceptable for older adults using services for the first time during the pandemic [20]. 

People already in receipt of services to alleviate loneliness and social isolation are arguably the most vulnerable to further loneliness and social isolation, so understanding the impacts of cocooning guidelines on such individuals is of utmost concern, both for service providers and those designing public health policy. As such, we conducted a study to understand the impact of cocooning measures and related adaptations to befriending services on people who had previously received the traditional, face-to-face format of such services. Our secondary aim was to develop recommendations for befriending services adapting to cocooning measures. We conducted a series of interviews with current service users between May 2020 and January 2021. During this period public health guidelines changed periodically in response to changes in COVID-19 case rates, but generally people aged 70 and over were advised to remain at home, avoiding any unnecessary social interactions. A qualitative approach was employed because of the exploratory nature of the research question and because our study represents a preliminary step towards understanding the impact of COVID-19 on befriending service users.

## 2. Materials and Methods

### 2.1. Design & Participants

A constructivist grounded theory design was used [21]. This design is suitable when the aim is to understand how a sample responds to a challenge, such as that posed to the receipt of befriending services during the COVID-19 pandemic. Sample size was determined a priori using the principle of information power [22] which considers five factors:

- Study aim (ours was specific, as we focused on the impact of public health guidelines on an existing befriending service, and on the recipients of such a service);
- Sample specificity (ours was dense, since participants were from a pool of individuals aged 70 and over, in receipt of a befriending service for at least one year, residing in the Dublin area of RoI);
- Established theory (no such theory was applicable here due to unprecedented nature of the challenge);
- Dialogue quality (assessed after completion of five interviews to be moderate, because of both tangential conversations and the learning curve of the interviewer);
- Analysis strategy (cross-case analysis conducted).

Taking these five factors into consideration, a provisional sample size of 10 participants, followed by an assessment of data saturation at this point, was determined. Participants were recruited from the population of existing befriending service users in ALONE who had been matched with their current befriender for at least one year. ALONE identified potential participants and asked if they would be interested in learning more about the research study. Potentially interested parties consented to have their details shared with the research team for follow-up. Ultimately, 11 participants were interviewed, and data saturation at this point was assessed and deemed satisfactory. Participants comprised three males and eight females (see Table 1). 

### 2.2. Measure

Constructivist grounded theory guidance on interview schedule construction was followed [21]. Four main questions, with sub-questions and probes therein, were developed (see Appendix A). Further refinements were made to the questions and probes following an initial pilot study with two participants in May 2020, and subsequently following each interview, as per grounded theory recommendations. 

### 2.3. Procedure and Ethics

Following identification of interested potential participants by ALONE, contact was made by the research team in the form of posted information sheets and consent forms, followed by a phone call after a period of one week. Participants were then brought through the consenting process by phone. If they agreed to participate, they were asked to sign and return consent forms by post. On receipt of these forms, the research team booked interview appointments with participants. All interviews took place by phone because of the cocooning guidelines in place. Interviews were all conducted by a trained research assistant (EH) under the supervision of an experienced grounded theory researcher (JMcHP). Supervision involved weekly discussions of all interview content, context, reaction of the research assistant, and necessary changes to the interview schedule. The average duration of interviews was 45 min. The study was approved by Maynooth University Social Research Ethics Committee (reference number 2401997). 

### 2.4. Data Analysis

Data were analysed according to constructivist grounded theory guidelines set out by Charmaz [21]. Audio recordings of all interviews made on an unnetworked Dictaphone were initially transcribed verbatim, and pseudonyms assigned to both the service user and their befriender as mentioned throughout the transcripts. Analysis was conducted after the transcription of each interview, and before the next interview was conducted. A process of initial coding was then followed, in which line-by-line segments of data were categorized and sorted. This process was followed by a period of focused coding in which codes were compared for analytic usefulness, retaining only those which provide a clearly comprehensive categorization of the most data. This phase was followed by the process of axial coding in which codes were compared to each other to understand their interrelations and how they may cohere under higher-order categories. Finally, these categories were examined and integrated to arrive at a grounded theory of managing with adaptations to cocooning and their impact on befriending services. 

## 3. Results

We describe a grounded theory entitled “maintaining personal autonomy” (see Table 2) in the face of the RoI government’s cocooning guidelines. While the government and ALONE both provided clear guidelines on avoiding unnecessary face-to-face contact during the pandemic, including that in the context of befriending services, participants in the current study adapted such advice to their own personal circumstances. Broadly, participants described two opposing (but often co-existing) states: *continuing with life as usual* (which at times meant contravening the government guidelines) and *adapting to cocooning* (with both positive and negative ramifications for wellbeing). These opposing states were *contextualized* by two further factors: trusting others to curtail the spread of COVID-19, and anxiety about personal risk. Ultimately, many participants spoke about *planning the future*, which included discussions about returning to life as normal, between lockdowns. In the final interviews, consideration of such a return to life as normal involved discussion of vaccination uptake. 

### 3.1. Category: Life as Usual

Some participants described making no or little changes to their behaviours in response to the pandemic, often contravening cocooning guidelines as a result. Others discussed how they saw themselves as living in a “pandemic-proof” way before the pandemic, and as a result had fewer adjustments to make. Some participants discussed explicitly the guidelines that they contravened. Overall, compliance to guidelines was high, but it was clear that such compliance was contingent on personal decision making—in this way, it was clear that participants maintained their sense of personal autonomy throughout the pandemic and associated guidelines. 

#### 3.1.1. Carrying On

Some participants reported that cocooning guidelines had not significantly impacted their lives. For many this was because they had already been living as though cocooning for years, due to health and mobility issues. Seán said: “It (cocooning) was pretty much the way I had been anyway”. Some participants spoke about the fact that social isolation specifically had, for them, been a state of affairs pre-dating the pandemic, as described by Saoirse: “This has made a big kind of change in all our lives, not so much mine…I wasn’t going out”. For these individuals, the guidelines were relatively easy to stick to, because it brought no change to their lives. Carrying on was related to an idea of already having been ‘pandemic-proof’—having lived in such a way that was analogous to cocooning before the pandemic. Being pandemic-proof meant staying at home and avoiding social contact, but it also meant getting essential goods via delivery instead of visiting the shops, and using careful preventive measures to avoid infection. Cait described: “Always before this pandemic I have little bottle with hand disinfectant gel”. The extent to which life remained unchanged, and to which participants could conceive of themselves as “carrying on”, was, for some, itself a coping mechanism. Carrying on was related to stoicism and getting on with life, as Seamus put it: “I carried on, you know? Positive outlook”. 

#### 3.1.2. Breaking the Rules

While participants described carrying on with their lives as normal during the pandemic, above, in ways that were almost incidentally compliant with the guidelines, they also described living as normal in ways that contravened guidelines. Carrying on as such could lead to compliant and non-compliant behaviours for this sample. While the sample were broadly living in ways that were compliant with cocooning guidelines, there were some behaviours that they had held on to which were in contravention of the guidelines. For instance, Brigid described: “No, I’m going to the post office, I’m going to mass”. Relating specifically to receipt of the befriending service, some participants also described “breaking the rules” (as Seán put it) here. This meant that they were carrying on or continuing with behaviours from pre-pandemic times, which were no longer advised. As Seamus stated: “I don’t think I missed a visit from V during the lockdown. He’d come same as he would anyway, well, anyway…I know V’s very clean and he wouldn’t come unless he was sure he was”. For Seamus, trusting his befriender to remain virus-free (“clean”) was the reasoning behind his continued visits. In this category, data suggested that participants felt comfortable in making their own decisions about behaviour during the pandemic, rather than strictly abiding by organizational or government guidelines. Mostly these decisions meant carrying on as usual, even if it meant contravening the guidelines.

### 3.2. Category: Adapting to COVID-19

Rather than describing consistency, mostly participants discussed the ways in which their lives had changed during cocooning. As well as adaptations to the befriending service. These changes were for the most part negative, framed as disruptions. However, participants also referred to some positive adaptations they had made to the situation. 

#### 3.2.1. Disrupting Life 

Most of the disruptions reported focused on reduced social engagement. For the most part, participants did not interact with others during cocooning. For instance, when asked whether she had seen anyone socially since the beginning of cocooning, Meadhbh said: “I observed it all, I wasn’t out, I followed the rules”. However, some participants continued to receive visitors during lockdowns, albeit with some adjustments made to minimise infection risk. This was not always a situation that participants themselves were comfortable with, however, as Saoirse described: “my niece came up last weekend, and she came in, and I couldn’t say not to come in because I wouldn’t do that, but sure we sat quite a long way apart from each other”. 

Disruptions were also reported to the way participants behaved outside of the home with many social and leisure activities halted. This was due both to the government-mandated closure of some services, like non-essential retail. In some cases it was due to participant reticence to expose themselves unnecessarily to risk of COVID19. As Meadhbh put it, “I love the shops…but I wouldn’t go now, obviously”. Padraig, who spoke at length about how he enjoyed meeting new people, commented on the difficulty that social distancing brought to this activity: “Summer is great, because you meet a lot of new people, you can sit out in the park, but with COVID you have to be keeping your distance”. As he described it, even relatively safe activities like socialising outdoors came with perceived risk that he was not prepared to take. Social and leisure activities were not the only ones to be disrupted, unfortunately. Participants also noted the disruptions they faced to more vital services, such as healthcare services and day centres. As Deirdre said: “I did do physiotherapy, she had me walking, but then because of COVID that went by the wayside”. For Deirdre this disruption meant that she had once more become effectively immobile during the pandemic, whereas before she had been working on rehabilitation. 

For some participants, the pandemic disrupted their plans in a broader sense, and made them rethink their living arrangements, and other requirements, such as private transport and holidays. Róisín, who had returned from Australia to Ireland during midlife, planned to return there to live with her daughter at the end of 2020: “With the coronavirus and that, I realised I need family”. The challenges of the pandemic had disrupted Róisín’s life to the extent that she was planning a considerable change in her life as a result. Since almost all participants who were interviewed lived alone, Christmas was a major concern in the interviews conducted in autumn and winter. Most of the concerns described were about having company on Christmas Day, and relatedly, having a Christmas dinner. The reasons given for concern over Christmas Day were not related to religion for anyone interviewed. Rather, participants had various concerns about their own health and that of relatives that threatened their plans to share the day, Padraig: “I always went to my sister’s for Christmas… I’m probably orientated, it might happen that I won’t be able to go”. It would seem that Christmas was an occasion that, even with the possibility of cocooning guidelines in place, participants were not prepared to spend alone. 

Many participants also felt that the disruptions they faced were more challenging in the winter than in summer, Gráinne: For two weeks I couldn’t get out, that drives me absolutely mad…after Christmas lockdown is more lonely than before, it’s freezing cold now nobody can go anywhere, that makes more depression I think”. Gráinne was unable to go for a walk during this period because of the hazardous icy conditions, and many participants expressed frustration in their last available outside activity, walking, being no longer available to them. Additionally, socialising, while relatively safe to do outdoors in the summer, was also less possible during the cold winter months. Padraig described: “In the summer months you could sit down where there was no COVID and you could talk to people…but now when the winter comes… your movements are restricted”. 

#### 3.2.2. Adapting to Cocooning

While many of the disruptions noted above were beyond the control of participants, many also reported that they had actively complied with cocooning guidelines, most notably as it related to their interactions with family and friends, and receipt of essential goods and services. Some of these adaptations were positive, and involved maintaining social interactions using the phone or video conferencing technologies. This was the case for Róisín, who, like other participants, took part in games with family members using videoconferencing: “During the lockdown, with my sister’s family, we do Zoom quizzes”. A significant adaptation made for some participants was to arrange deliveries of essential goods, either from businesses or by family members, as was the case for Seamus: “Another son comes with the groceries”. Almost all participants had either already been receiving groceries in this way, or switched to doing so during the pandemic. This was despite, during the second and third lockdowns, supermarkets making available specific times in the day for older or medically vulnerable individuals—while this option was available to participants, they preferred to have their groceries delivered. 

Adaptations were also made in relation to essential services, e.g., the receipt of home or healthcare, which for many participants was a source of anxiety, for example, Brigid, who had homecare once a day: “I don’t go out that much but anyone that comes in has a mask”. Gráinne had a particular issue getting to the hospital without help, as due to a spinal injury she had little mobility: “Before the corona a friend of mine used to take me and bring me back…now I have nobody to go to the doctor’s so sometimes I say right, I have nobody so I have to do something, so I get the taxi, if I fall taximan is there, if I’m going to hospital, there’s a security man, so I just go and do it!” Participants had to make adaptations to their receipt of services, but mostly felt anxious in receiving these services despite the adaptations made. Gráinne’s story about visiting the hospital alone in the face of mobility issues made clear that she taking significant risks (of falling) in order to receive essential services.

#### 3.2.3. Disrupted Befriending Services

The adaptations necessary during cocooning specifically impacted the befriending services typically received by participants, and they spoke at length about such disruptions. Some of the changes could be framed as interruptions, while some were adaptations. For many, befrienders took on additional instrumental roles during the lockdown. Most participants reported that during lockdown, their befrienders did not visit them, and that they were satisfied with this decision. Seán stated: “It wouldn’t be proper for us to be meeting anyway, because I’m still supposed to be cocooning”. 

Some participants reflected on the activities that they used to share with their befrienders, which had been interrupted by the pandemic. Participants on the whole reported that they missed these shared activities. Padraig described missing group events with his befriender particularly: “the ALONE functions are not taking place now, which is a great pity”. 

Participants were asked specifically about their attitudes towards phone-based befriending, since this was the suggestion made by ALONE to replace visits during cocooning. Most participants saw this as an inferior but acceptable alternative to face-to-face befriending: Meadhbh: “(Befriender) rang me every Thursday, she said if you’ve any problem in between ring me, so we’re in contact the whole time, that was good”. For others, phone calls remained an instrumental device to arrange visits or solve practical problems, Seán: “I’d say I think a few times I might have rung him about something practical, you know, a particular need I had, but no, generally speaking, it was all practical things”. It appeared that for some participants, phone calls could be used to take the place of visits, allowing simple social contact. For others, phone calls were more instrumental and less social. The interviewer also asked participants about whether they would be open to using video calls during lockdown to connect with their befrienders, but mostly, participants did not like this idea, such as Ailbhe: “No no no I couldn’t do that, no”. 

Adjustments were made by some to allow the continuation of the befriending relationship. For instance, Ailbhe continued to see her befriender in her home, but with infection control measures in place: “The last time [befriender] was here we wore masks”. Others did this too, leaving windows open and using hand gel but broadly continuing with face-to-face befriending visits. Some participants, during the summer particularly, continued to meet their befriender, but outdoors, or at the door, to reduce risk of infection. As Róisín put it: “[Befriender] came during lockdown, she would sit outside the door, and I would sit inside the door and we’d have a chat”. Not all participants saw the value in making such adjustments. For instance, Deirdre: “I haven’t seen [befriender] at all since summertime…there’s no point ringing her to stand outside the wall and shout in, it’s ridiculous really, no I haven’t kept in touch with her really”.

It became clear during interviews that some participants regarded maintaining the social and relational aspects of the befriending relationship as key. For others, more practical matters, especially relating to shopping, supplanted the typically social nature of the relationship, Cait: “[Befriender] helped me, she bring me this time to get more food…sometimes she has a telephone conversation, she say what you want cos I will bring”. Such practical supports were critical for many participants, who had lost other supports during this time, both from services and from family. For instance, Padraig told of his recent hospitalisation, during which family members could not, for their own health reasons, visit him: “[Befriender] stepped in, right away…he said, if there’s anything I can do for you, he said don’t hesitate…the hospital were not able to look after cleaning my clothes so I had to ask [befriender]…he went to my sister numerous times and brought all my fresh clothes to me. He also from time to time came to the hospital reception and left some snacks for me and magazines”. In this description, Padraig demonstrates the critical role that his befriender had played in his health and wellbeing during the pandemic, despite being a voluntary and discretionary contact. As well as receiving instrumental support from befrienders, participants also reported receiving informational support, during a time when there was a lot of mistrust and misinformation about the pandemic and vaccinations, as reported by Meadhbh: “[Befriender] keeps me up to date on a lot of things, what’s going on in the world…she’s very informative, if we’re talking about COVID, she’d have a different view…I do think she’s very informative”. 

As well as adaptations made by befrienders and participants further adaptations had been made at an organisational level in response to the pandemic, and as such participants reported that they had been offered additional services from ALONE. Ailbhe in particular appreciated a poetry activity offered by ALONE during the pandemic: “ALONE have been excellent now since the COVID…they said they’re doing this poetry reading thing or something, and she said I thought you’d like that, and I loved it!”. These additional services, and offers made by ALONE were much appreciated by participants. Participants reported that they had been offered additional phone calls and other supports, which many agreed to receive. Seamus among others, however, felt that he did not require such additional service, and in fact felt that it would be taking services away from people in need if he were to accept: “I felt that ALONE were under a lot of pressure…during the COVID I was asked, they said like will they phone me, and I said look it, you have a lot of work to do…there are a lot of people out there who would appreciate it more so, that would need it more so than I”. Participants made clear that they appreciated and valued the services offered by ALONE during the pandemic, and that they wished for these services to go to those most in need. 

### 3.3. Context

As described above, participants referred to changes in their lives as a result of the pandemic. These changes were further contextualised during interviews, both by societal trust, and by participants’ own personal circumstances. Societal trust was described in relation to the actions of others around them, and in relation to trust in the government’s handling of the pandemic. Personal circumstances, specifically participants’ feelings of anxiety or specific health concerns concerning COVID-19, also shaped their response to and compliance with the cocooning guidelines. 

#### 3.3.1. Trusting Others

Participants discussed their trust in others around them, and their trust in the government. Participants framed both groups as being critical in keeping them safe, and had mixed feelings about the extent to which they could be trusted to do so. Some participants perceived that others around them were complying with the government guidelines, e.g., Saoirse: “The area is not a very busy area but it’s more than quiet now, cos nobody is out…the thing about it is if somebody gets out of line, it’s noticed”. More participants, however, felt that others were not doing their part to prevent infection. As Róisín put it: “I mean people don’t wear the mask, you know, I’ve been to the post office twice, and nobody wearing the mask in there either”. Trust was also evident in the manner in which some participants referred to the government response to COVID-19 more generally, e.g., Seán: “I must say for 2/3 of the period of lockdown, I was very positive about the government’s response to the crisis, I thought they played a blinder”. While Seán’s description of the government response here is mixed, many other participants were far more critical of the government response to the pandemic. This was stated bluntly by Deirdre: “I have no faith in the government”. Overall, participants were distrustful of those around them, particularly the government, to keep them safe during the pandemic. 

#### 3.3.2. Anxiety about Personal Risk

Decisions about adapting to the pandemic were also made in the context of personal risk. Participants felt anxious about many aspects of the pandemic: they expressed fear of catching COVID-19, and the fear involved for one participant when he caught COVID-19. Some participants viewed their personal risk of becoming seriously ill with COVID-19 as very high, and these participants were therefore more vigilant in reducing their risk of contracting COVID-19, as was the case with Seamus: “I’ve only half a lung, so I would fear it more than anything else”. Padraig felt anxious about COVID-19 specifically because he had already contracted it while convalescing at a nursing home. He described the experience: “I had to go into isolation for 14 days, the 14 days’ isolation was like 14 years…it was frightening, you know”. This anxiety had impacts on participants’ behaviour during the pandemic. For most, this meant that they avoided supermarkets and other crowded places. However, more worryingly, Gráinne reported that she was avoiding hospital appointments because of her fear of catching COVID-19 there: “they asked me to come to hospital to do the bloods, I say I’m not coming until the end of March…because I don’t have COVID, I don’t want to go and get something”. 

### 3.4. Planning the Future

As well as reflecting on the changes they had and had not made in response to the pandemic and cocooning guidelines, participants also spoke of looking forward to the future and the return to normal that they hoped this would bring. Some respite was experienced in the periods between lockdowns during 2020, and participants commented on the positive impacts that these respites had on their lives. Participants identified specific activities that they looked forward to once the pandemic ended. Some of the interviews were conducted after reports of successful vaccination trials had entered the media. As such, for some, the return to normality became predicated upon receiving a vaccine. However, for some, anxiety about the effects of such a vaccine remained. 

#### 3.4.1. Returning to Life as Normal 

Some participants were specifically were looking forward to the return of their befriender visits. Some participants spoke about an ultimate return to befriending visits, coming at the end of the pandemic. Others spoke about the resumption of befriending visits that they had during reprieves between lockdowns, when cocooning guidelines in RoI were relaxed. Roisín spoke about feeling excited to see her befriender after the first lockdown: “When ALONE contacted her to say that they could resume the visits, she was delighted, as was I, and the very first week she was back and we had social distancing in the house and we shared a cup of tea and a biscuit together”. 

Other participants appreciated returning to other activities both between lockdowns and ultimately at the end of the pandemic. These activities varied across participants. For Seán and others, shopping was important: “Shopping was a big deal, I was doing my own shopping right up to the coronavirus and I’m back doing it now again”. Shopping (for groceries) was raised in many interviews, not just because it was so critical to survival, but because as Seán put it, it was a “big deal” and connoted independence. For some participants, hairdressing services were a major concern: Saoirse: “The one thing that I, we, are all mad [keen] to get open is the hairdressers, the hairdressers is a big problem for the likes of me now”. 

For Seamus, the return to normality meant being able to see his grandchildren, “I have my grandchildren come to me, I know it was a while before I got to see them, like, you know, I couldn’t see them for a good while then. They’re able to come and see me now so that’s a good thing too”. For Roisín, easing restrictions meant being able to travel beyond the 5 km radius implemented as a limit during lockdowns: “The 5th of June, I think it was, when they said people could go 5 km, and I got in my car and I drove down exactly 5 km to the sea, it was lovely, the sea and the wind, aw it was paradise, I made a short video which I sent to all my friends saying “freedom at last!”” 

Part of the impetus to return to normal was to remove perceived burden from befrienders and others who were supporting the participants. Cait described how she often went without essential foods such as milk. With her only family abroad, the only person Cait had in her life who could help her with groceries was her befriender, and she did not want to burden her with requests: “I not disturb [befriender] also because she has a family, she working person also”. 

#### 3.4.2. Vaccination

From November 2020, there was increased coverage of vaccination trials in the media and this became a topic of interest during the five interviews conducted after this point. Cait had decided to avoid vaccination because she believed that it would make her sick: “My daughter is doctor, and she I promise that I not take any flu injections every single autumn, because they putting inside bacteria, and you get sick …I promise that I not take and she promise me that she not take, because keep your body, clean hands, and eat natural”. While Cait’s attitude to vaccination was quite extreme, many other participants expressed milder concern about the potential side effects of vaccination. Part of this concern arose because the vaccinations had been developed in such a short space of time. As Meadhbh put it: “there isn’t enough time to see what’s going to come down the road”. Other participants, however, were very eager to receive the vaccination. This was described by Gráinne: “the HSE are going to get in touch with me for the injections, so I’m waiting for the COVID-19 injection, vaccine…I’m going to do it, everybody should get it”. Only two of the five participants had polarised attitudes towards taking the vaccination (either positive or negative); the other three spoke about their mixed feelings, because of concerns to do with vaccine safety, but all three reported that they would likely receive the vaccination. 

## 4. Discussion

We explored the experience of cocooning among individuals who typically use befriending services, since such individuals may be at increased risk under cocooning guidelines (2). We also aimed to identify acceptable adaptations that could be recommended to befriending services. We present a grounded theory of “maintaining personal autonomy” in the face of cocooning guidelines issued by the RoI government during 2020 and 2021. Maintaining or carrying on was a theme described by many participants, both because they had already been living in a pandemic-compatible way, and because carrying on appeared to denote a stoicism in the face of a challenge. It appeared that for most participants, carrying on with life was of primary importance, with cocooning guidelines secondary to this. Participants were, on the whole, compliant with government guidelines, but contravened these guidelines as it suited their circumstances, for example, to receive visits, to continue with face-to-face befriending, and to do their own shopping. Participants described their behaviour and compliance in the context of their own, self-assessed risk in relation to contracting COVID-19 and becoming sick as a result. Participants discussed at length the disruptions that the pandemic had brought to their lives but also the ways in which they had adjusted their lives to the pandemic. 

In relation to compliance, a minority of participants indicated that their lives had not changed at all because of the pandemic—most reported that they had adjusted to the pandemic and the cocooning guidelines. This finding is compatible with previous reports that older adults are relatively protected from the negative mental health effects of the pandemic [15,16], a finding which may be explainable with recourse to models such as the “strength and vulnerability integration” (SAVI) approach [23], or the selective optimization with compensation model of preserved wellbeing in later life [24]. According to these approaches, older adults strategise to maximise positive affect in later life, and minimize the experience of negative affect. As would be expected based on these models, few participants in our study reported that the pandemic had an impact on their wellbeing. However, all participants indicated that the situation was not ideal and had a preference for returning to life as normal, particularly as that related to their receipt of befriending visits. Some participants’ lives had not been significantly impacted by the pandemic—because they were already spending most of their time at home and receiving instrumental support from others in the form of deliveries and shopping. This insight may in part explain why this sample appear more robust than younger adults to the isolating effects of COVID-19—their levels of isolation and confinement pre-dated the pandemic, and so was less atypical. It may also explain why this sample differ to the broader population aged 60+ who were recently surveyed and reported increases in loneliness and depression (7,8)—for our sample, cocooning may not have brought about as much change to their lives. Another possibility is that our sample were experiencing stable levels of emotional loneliness which were not affected by the pandemic, rather than social loneliness, which would theoretically be more susceptible to the effects of social isolation [25]. TILDA, the study described earlier, measures loneliness using a unidimensional approach (8). Measuring both emotional and social loneliness and their trajectories during cocooning, at a population level, would help us to understand the specific aspects of loneliness most affected during such a time.

A minority of participants too indicated that they contravened the rules or guidelines on cocooning during the pandemic and mostly in relation to necessary visits and services. This finding would suggest that the guidelines were not fit for purpose. In subsequent lockdowns, changes were made to the guidelines, including the introduction of safe times for shopping for people aged 70 and over, and “support bubbles”, whereby a household of an older adult living alone could pair with another household for instrumental and emotional support. This finding also suggests that while government guidelines are based on protecting the physical health of people aged 70 and over, they do not (or did not at the beginning of the pandemic) take into account social and emotional wellbeing. In order to have the maximum compliance possible, guidelines related to the wellbeing of older adults should consider these facets of wellbeing also, and cater for them. Effective government guidelines would also consider the critical role of the voluntary sector in maintaining the wellbeing of older adults. 

Our results indicate that befriending services play a critical and often practical role in the lives of older service users, and such services continued to be delivered during the pandemic on the basis of the relationship already formed between the service user and their befrienders. For instance, Cait recounted how her befriender would buy her extra groceries when doing her shopping—a form of practical support she appreciated. This cost is something that could be catered for. Formalizing and formally acknowledging the contribution of befrienders to the lives of service users during the pandemic would allow the directing of critical resources to support these relationships. This cost is something that could be catered for in a similar vein to action taken by the Scottish Government in their Befriending Fund announced in December 2020 (befriending.co.uk/news/new-funding/, accessed on 1 December 2020). While the aim of a befriending service is to establish companionate bonds, which many users characterise as friendships, transgressing the boundaries of such a structured relationship can lead to feelings of obligation which can threaten the relationship [26]. As such, it is critical that services ensure their befriending volunteers continue to be supported if they choose to transgress such boundaries and provide additional support to service users during extraordinary events such as a pandemic, or indeed during other events such as illness or hospitalisation.

It was of interest that some participants described mistrusting other people when it came to following social distancing guidelines—meanwhile, participants appeared to trust their self assessments of risk and behaviour, as it allowed them to maintain personal autonomy in a context of relatively strict governance. Much has already been written about trust in the context of COVID-19—both of others and of the government response. Interestingly, individuals who are highest in general social trust are most likely to engage in preventive behaviours in the context of COVID-19 [27] and in compliance to public health measures during the H1N1 influenza epidemic [28]. As such, it is logical that participants would demonstrate a lack of trust in others concomitant with non-compliance to all cocooning guidelines. It would be of interest to explore further whether mistrust was felt of the befriending services also—in the case of two participants, mention was made of a lack of compliance with ALONE’s guidelines. Evaluating or promoting organisational trust would be important for any organisation delivering befriending services, since it would likely increase compliance in organisational measures. 

Participants described changes made to their lives as being either acceptable adaptations, such as receiving deliveries instead of going to the pharmacy, or unacceptable disruptions, such as the inability to have visitors to the home, for some. Many technological solutions have been proposed to assist older adults facing social distancing measures during the COVID-19 pandemic [29]. While previous research showed that switching to other channels of communication (phone, videocalls) was acceptable [20], our sample framed these channels as inferior to face-to-face visits. In Rodenstein’s paper, however, the service was new to users, while our sample had previously experienced face-to-face befriending and as such had a comparison to make. Other recent research focusing on continuation of befriending visits for nursing home residents using remote options has emphasised the need for the befriender and service user to work collaboratively on identifying a communicative option they both accepted [30]. This emphasis on collaborative decision making should be made by befriending services serving older adults living independently too. 

Vaccinations were discussed by five of the eleven participants interviewed. Of these five, four raised concerns about receiving the vaccine. Such concerns recently been shown to be present in about 1/3 of a sample of the RoI population and appear to be linked to informational receipt from non-traditional sources and with mistrust [31]. Murphy and colleagues argue that intervening on such vaccine hesitancy could be done by delivering information about vaccination via non-traditional and trusted sources, which could potentially include befrienders. This informational support was alluded to by one participant, Meadhbh, who alluded her befriender bringing information about scientific developments in relation to COVID-19. It is possible that befriending services may not wish to implicate themselves in encouragement of vaccinations to their service users, but this may represent a critical path in promoting vaccine uptake among such individuals. 

Significant changes to the receipt of befriending services were also discussed—while it is laudable that so many befrienders took on additional support roles necessary for participants during the pandemic, the clear danger presented in these interviews was that befriending services for some became entirely instrumental. The aim of befriending services is to provide social and emotional support for individuals, rather than practical supports—it is clear that for some, such support became deprioritised during the pandemic. It would be a concern for befriending services to ensure that a return to the delivery of social and emotional support is made as soon as is practicable. Additional to this issue is the clear requirement for practical supports on the part of the service users. It may be more appropriate to link service users with other services dedicated to supporting such practical requirements. ALONE and other organisations already use a ‘support coordination’ model to integrate supports for service users, so perhaps such services could support the additional roles taken by befrienders during the pandemic, in a more formal manner. 

Findings were considered in relation to their potential applicability to services offering befriending to older adults during the COVID-19 pandemic, and from the above data, seven recommendations for practice were made. 

### Recommendations for Befriending Services

Based on the interviews conducted, seven recommendations can be made to befriending services interested in adapting their services for the pandemic:

1. Switching to phone-based befriending is only a solution for some service users: the focus should be on collaborative decision making about communicative alternatives for each befriender-service user pair.
2. Many participants reported that their befriender had taken on additional, more practical roles during the COVID-19 pandemic. Notably, the absence of face-to-face contact and dominance of phone-based contact appeared to, for some, mean that the social and emotional aspects of the befriending relationship were neglected—the phone represented a means of communicating about practical matters only. It is worth evaluating whether users and befrienders alike feel that this switch has deprived them of social and emotional contact. If pairs are happy to continue their social contact via phone, this may be a good solution. If pairs report that their relationship has become purely practical, it may be more appropriate to separate practical supports from social and emotional supports from an organisational perspective—possibly as supported by the existing “support coordination” model of care.
3. Participants were not interested in switching to videoconferencing facilities. As such phone-based befriending may be the only acceptable alternative for this group. However, it should be noted that the group interviewed were in the “older old” category and this preference is likely to change with subsequent cohorts.
4. Participants felt the absence of group-based activities often run by ALONE. Depending on community rates of COVID-19, it may be possible to organise small-group outdoors activities for service users who remain interested in meeting other service users.
5. Some participants expressed mistrust in others, including in the organisation, in relation to cocooning measures. Evaluating organisational trust in a structured manner and working to increase it would likely improve compliance in organisational measures in response to the public health governmental guidelines.
6. Many participants felt that their befriender was the last service available to them, which may have placed stress or pressure on these befrienders at a time when they were likely already under strain related to the pandemic. Some participants were cognisant of this and did not want to add to the burden of befrienders. It would be important to check in with befrienders and provide alternatives to ensure that service users are well supported but not at the (personal or financial) cost of befrienders to avoid undue transgression of relational boundaries.
7. Befriending services may need to consider their role, if any, in promoting vaccination uptake among service users, potentially via befrienders. While this is not an adaptation to the service per se, improving vaccination uptake would curtail the duration and severity of the COVID-19 pandemic at a societal level as well as maintaining the wellbeing of service users, whose health and wellbeing is the main concern of such services.

Such recommendations may help befriending services to adapt to the ongoing pandemic and associated cocooning measures. Further research would be required to evaluate the effectiveness of such recommendations, and to further evaluate the psychological impact of cocooning measures and service adaptations to those typically in receipt of befriending services. 

## 5. Conclusions

The study aimed to evaluate the impact of cocooning measures on RoI recipients of a befriending service and understand how such services could be acceptably adapted to comply with such measures. We developed a theory of “maintaining personal autonomy” in the face of cocooning measures, and developed practice recommendations for befriending services based on findings. While compliance among befriending service users was generally high it was not total. There may be ways in which befriending services and befrienders can adapt their services to better support users during this challenging time, as well as leveraging their provision of informational support to improve the health and wellbeing of service users by encouraging compliance and vaccine uptake. 

## Figures and Tables

**Table 1:** Sample characteristics of the 11 study participants involved in grounded theory interviews.

Pseudonym	Sex	Age	Living Situation	Date of Interview
Saoirse	Female	85	Living alone (in house)	6 May 2020; Cocooning/Lockdown *
Seán	Male	73	Living alone (in apartment)	22 May 2020; Phase 2
Róisín	Female	81	Living alone (in house)	7 July 2020; Phase 2
Seamus	Male	76	Living alone (in house)	4 October 2020; Level 3
Padraig	Male	78	Living alone (in apartment)	5 November 2020; Level 5
Deirdre	Female	71	Living alone (in house)	25 January 2021; Level 5
Grainne	Female	69	Living alone (in apartment)	19 January 2021; Level 5
Meadhbh	Female	79	Living alone (in house)	17 December 2020; Level 3
Brigid	Female	96	Living alone (in house)	15 December 2020; Level 3
Cait	Female	72	Living alone (in apartment)	8 December 2020; Level 3
Ailbhe	Female	81	Living with daughter (in house)	20 January 2021; Level 5

* RoI cocooning measures came into effect on 12 March 2020; people aged 70 and over were advised to stay at home (“cocoon”) and avoid any unnecessary social contact. From 18 May until 8 June 2020, Phase 1 of the “roadmap for reopening society and business” began, during which cocooning advice remained active. From 5 June 2020, Phase 2 of the roadmap was in effect, during which people aged 70 and over were advised to remain home as much as possible, to practice social distancing with visitors to the home, and shop at specific times allocated by retailers. A new plan was published on 15 September 2020 with a change in government, and Level 2 restrictions came into effect, in which mandatory face coverings and altered recommendations for home visits, but otherwise no change to the previous plan for those aged 70 and over specifically. Level 5 was introduced to the country on 21 October 2020 until 8 December 2020, when all restrictions were relaxed for the Christmas period, leading to a “third wave” of COVID-19 infections and subsequent Level 5 lockdown, in which all citizens were advised to remain at home and avoid unnecessary journeys and social contact, began on 31 December 2020.

**Table 2:** Categories and subcategories/themes presented in the grounded theory “maintaining personal autonomy”.

Category	Subcategory/Theme	Example Data
Life as Usual	Carrying on	P012: “You have to push yourself…it’s hard for a lot of people now to keep going”
	Breaking the rules	P008: “I had visits, you know, from family like…nothing’s changed, you know?”
Adapting to COVID-19	Disrupting life	P009: “I attend a day care service three days a week and they’ve been stopped now with COVID, so basically I’ve only L now, of all the services I had, L is the only one left”
	Adapting to cocooning	P012: “I did a lot more cooking, baking, and sat out in the sun in the garden”
	Disrupted Befriending Services	P007: “We went to the movies in that other world when we were allowed to do those things”
Context	Trusting others	P011: “I say to all the kids, who are 18,19, I keep saying where is your mask? As they passing by, please wear the masks because you don’t know if you have it or if I have it”
	Anxiety about personal risk	P005: “I think of it because my age a lot…well if I got it, I’m sure I wouldn’t be strong enough, I’d get sick, you know, some people seem to get over it very quickly but I don’t know that I would…it’s the most alarming thing, really”
Planning the future	Returning to life as normal	P005: “Well, I’d say they’ll have to start saying that people can come and go in people’s houses, so maybe there’s some little light at the end of the tunnel”.
	Vaccinations	P010: “I feel awful saying this but like vaccinating a woman of 100, before a doctor who is in that awful atmosphere all day in plastic and head gear, that I can’t understand”

## Appendix A. Interview Schedule

1. Firstly, are you still observing the cocooning/social distancing guidelines as set out by the Department of Health (update as appropriate)?
  - What do you think about the Department’s response to the pandemic?
2. Can you tell me about the impact that these guidelines are having on your day-to-day?
  - Are the changes making you feel isolated? Are they making you feel lonely? /lonelier than before?
  - Can you tell me about your own personal risk in relation to COVID-19?
3. In March, when guidelines were issued:
  - Did you stop receiving visits from your befriender?
    - (if stopped) whose idea was it to stop visits?
    - (if continued) whose idea was it to continue visits?
  - Did you receive guidance from ALONE directly on applying the guidelines?
    - Would you say you needed more guidance than you received?
  - Would you have continued the visits (against guidelines) if your befriender was willing to do so?
4. It looks like the pandemic will change our society for longer than expected. Thinking about the rest of 2020/2021, then, how do you think it will impact your visits from your befriender?
  - For how long do you expect these changes to affect your visits?
  - Under what circumstances would you be happy to have visits again?
    - What measures would need to come into effect?
  - If no visits are permitted until next year, what would you like as a replacement until then?
    - Phone calls? How frequent? From your befriender/someone else?
    - Outdoor calls (e.g. calls to the garden)?
    - Skype or videoconferencing?
    - Would you be happy if ALONE started using technology to contact you, e.g. video conferencing? What if this replaced visits?
